# Acknowledgment to Reviewers of *Behavioral Sciences* in 2020

**DOI:** 10.3390/bs11020017

**Published:** 2021-01-26

**Authors:** Behavioral Sciences Editorial Office

**Affiliations:** MDPI AG, St. Alban-Anlage 66, 4052 Basel, Switzerland

Peer review is the driving force of journal development, and reviewers are gatekeepers who ensure that *Behavioral Sciences* maintains its standards for the high quality of its published papers. Thanks to the cooperation of our reviewers, in 2020, the median time to first decision was 17.8 days and the median time to publication was 3.7 days. The editors would like to express their sincere gratitude to the following reviewers for their precious time and dedication, regardless of whether the papers were finally published:
Abad Robles, Manuel TomásLeyton Roman, MartaAbate, GiuliaLim, Kam MingAbreu, Ana MariaLima, MartaAdam, MarcusLin, Pei-KuanAgbangla, NounagnonLiutsko, LiudmilaAgostini, AlexandraLongo, StefanoAlarcon, GracielaLópez Díaz, Alfonso IsidroAlmendra-Pegueros, RafaelLopez, AntonellaAlonso, FranciscoLopez-Villegas, AntonioAlrahaili, MusaadŁukowski, AdrianAmor González, Antonio ManuelLunagariya, AbhishekAmutio, AlbertoLundberg, Ulf L.Anastasiu, LiviaLuo, HanAndrade Cetto, AdolfoMaciaszek, JanuszAndriessen, KarlMadan, PavanAntoñanzas, JoseMadariaga, AuroraAparisi, DavidMajor, Sofia O.Arcos Romero, Ana IsabelMalinowska-Cieślik, MartaArmaou, MariaMalone, PamelaAsonitou, SofiaMandić-Rajčević, StefanAssari, ShervinMandolesi, SerenaAyuso, Juan MielgoMansourian, SuzanBadau, AdelaMarcassa, StefaniaBadulescu, DanielMarchena, CarlosBagley, Christopher AdamMarek, AgnieszkaBaldwin, Ann L.Marques-Sanchez, PilarBaldwin, Paula KMarsh, Kerry L.Ballmann, ChristopherMartín, Jesús SánchezBaran, JoannaMartinez Ferreiro, SilviaBarbier-Greenland, KatyMartínez, Juan PedroBarkauskienė, RasaMartini, DanielaBarkley-Levenson, AmandaMathisen, Therese FostervoldBarrios, MaiteMatud, PilarBarros, SílviaMauriello, FilomenaBaudson, Tanja GabrieleMaurya, ShailendraBaumgarth, William P.Mavrou, IriniBayram-Jacobs, DurdaneMazilescu, Crisanta-AlinaBeaglehole, BenMazoteras-Pardo, VictoriaBecker, NicolasMazur, JoannaBelford, NishMcBride, AnneBenesh, JulieMcClement, SusanBerg, KristenMcDougall, JulianBernárdez-Vilaboa, RicardoMcGill, Ryan J.Birau, Felicia RamonaMchill, AndrewBlasco-Ros, ConcepciónMedová, JankaBobkina, JelenaMeerza, Syed Imran AliBogdan, MariaMendez Santos, Maria Del CarmenBoggiano, Victoria L.Mesko, MajaBollig, GeorgMessineo, LudovicoBonini, Sara AnnaMestre, Jose M.Borawska, AnnaMichaeli, MotiBorg, IngwerMiguel-Revilla, DiegoBorzikowsky, ChristophMihaicuta, StefanBottini, GabriellaMileti, IlariaBozzato, PaoloMilić, JakovBradshaw, NicholasMolero Jurado, María Del MarBrand, SergeMolodchik, Mariia A.Breeze, RuthMond, JonathanBronkowska, MonikaMonsuez, Jean-JacquesBrown, ClareMoody, KyleBrown, EmilyMoret-Tatay, CarmenBuckley, JeffreyMorinaj, JuliaBudhrani-Shani, PinkyMouton, BénédicteBuksnyte–Marmiene, LoretaMucci, NicolaBurghardt, KyleMurff, Sharon H.Burgos-Garcia, AntonioMurillo, Marisol LilaBurlacu, SorinMustafa, GhulamByeon, HaewonMyszkowska-Ryciak, JoannaCáceres Reche, PilarNash, CarolCaffò, AlessandroNatale, VincenzoCalabria, MarcoNavarro, RaulCaldeiro Pedreira, Mari CarmenNeiva, Henrique P.Calvo, Ana CristinaNewsome, CheyenneCamacho, David ParraNewson, LisaCamp, MargaretNg, Qin XiangCañabate, DolorsNjoku, AnuliCañadas-De La Fuente, Guillermo A.Nomura, KyokoCantero-Garlito, Pablo A.Olivares-Olivares, Pablo JoséCaparros-Gonzalez, Rafael A.Oliveira, CidaliaCapitman, John AmsonOlmos Gómez, María Del CarmenCapuano, AngelaOlszewska-Guizzo, AgnieszkaCarlini, Célia R.Onofrei, Roxana RamonaCaron, RosemaryOrtega-Campos, ElenaCarrozzino, DaniloOzamiz-Etxebarria, NaiaraCasanovas, CarmenOzbič, MartinaCastelli, VanessaPardo, RebecaCastro, Maria AntónioParibello, PasqualeCava, María-JesúsPasquini, MassimoCavallo, MarcoPastorino, Grazia Maria GiovannaCenik, BasarPathak, DhrubaCerniglia, LucaPatra, AbhijeetChakraborty, DarpanPech, MartinChamine, IrinaPeletidi, AlikiChang, Yao-LangPellas, NikolaosChang, Yun-HsuanPentaris, PanagiotisChapple, HelenPerez, FranciscoChen, BoPerez, Miguel A.Chen, ChongPérez-González, CarlosChen, FengPeterson, David A. M.Chen, Yu-ChunPetrinovic, Marija-MagdalenaChiew Hong, NgPeyton, Joy KreeftCimino, SilviaPiñeiro Aguín, IsabelCittà, SantinaPirchio, SabineClaros, EdithPisanu, ClaudiaCodina, NuriaPolanska, KingaCohen, DanPolanski, WitoldCollie, CraigPollini, AlessandroComeau, KatiePonto, Maria T.Conejero, AngelaPoole, JayConnor, MelaniePortilla-Tamarit, IreneConstantinou, Costas S.Pourié, GrégoryCox, Christopher R.Pozo-Rico, TeresaCushing, SharonQin, SanboCzarniecka-Skubina, EwaQuek, TianDamiani, PaolaQuevedo-Blasco, RaúlDando, CoralQuide, YannDe Berardis, DomenicoQuraishi, MuzammilDe Bruin, AngelaRadzimski, AdamDe Gennaro, LuigiRahman, MahfuzurDe Juanas, AngelRamachandran, RoshiniDe La Torre, Gabriel G.Rapoport, David M.De Santis, NicolettaRapp, PaulDe Stefano, RosaReybrouck, MarkDelgado, GozaloReznik, Jacqueline E.Di Battista, SilviaRhodes, Diane McDanielDi Giacomo, DinaRicci, FedericoDi Maggio, IlariaRoccella, MicheleDiaz, AnjoliiRodríguez, Carlos FreireDíaz, Estela M.Rodriguez, KerriDíaz-Meneses, GonzaloRogobete, Alexandru FlorinDillon, DeniseRogoza, RadosławDoddapattar, PrakashRomero Martínez, ÁngelDombrovskis, ValerisRoskos, BeverlyDore, BettyRowley, GeorgiaDöring, NicolaRoy, BidishaDorocki, SławomirRuggieri, Ruggero AndrisanoDos Santos, Luis MiguelRusu, Alina SimonaDrzazga, ZofiaRyan, AnthonyDuarte, PauloSá, Thaís DeDubiel, BozenaSaad, MarceloDueñas, Jorge-ManuelSaarelainen, Suvi-MariaDumitrache, LilianaSabharwal, PriyankaDundes, LaurenSadowski, JerzyElena, Bernabéu-BrotónsSáenz-Lopez, PedroEmmitt, StephenSah, NirnathEnglund, TessaSalucci, SaraEsangbedo, Moses OlabheleSamul, JoannaEspejo, BegoñaSánchez Sánchez, Laura Del CarmenEwert, AlanSanchez-Gomez, MartinFang, Mei LanSánchez-Rico, MarinaFein, GeorgeSandham, AlexandraFernández-Prados, Juan SebastiánSantha, AgnesFernández-Revelles, Andrés B.Santric Milicevic, MilenaFeu, SebastianSanz-Paris, AlejandroFigley, Charles R.Sar, Bibhuti K.Fincham, AndrewSarapultsev, AlexeyFlorán, BenjamínSauter, MarianForte, GiuseppeScandurra, CristianoFoster, ElissaSchermer, Julie AitkenFox, Claire L.Schmitz, FlorianFreedman, RobertSchubert, Anna-LenaFreeman, Max R.Schumm, Walter R.Fucà, RominaSediqzadah, SaadiaFudge, NinaSemu, Linda L.Gade, MiriamServidio, Rocco CarmineGagné, IsaacSettembre Blundo, DavideGallardo Vázquez, DoloresShan, Xiaojun (Gene)Gamble, Jeffrey HughShang, GuowenGander, FabianShelley, MackGarcía Buades, María EstherShelley-Tremblay, JohnGarcia, AmberShen, JunGarcía, OscarShih, Hao YuGarcía-Carrión, RocíoShintel, HadasGarcía-González, JessicaShiwaku, HirokiGarrett, Mario D.Sierra, Javier OrtuñoGash, HughSil, SusmitaGeorgian, BadicuSimos, JoannaGheorghe, HuzaSingh, ChanpreetGiannakeas, NikolaosSiniscalco, DarioGibbons, JeffSivrikova, NadezhdaGibuła-Tarłowska, EwaŚlaski, SławomirGil Madrona, PedroSnauwaert, Dale T.Giuliani, FrancescaSobba, Kristen N.Goldsmith, KimberleySoreca, IsabellaGómez-Urquiza, Jose L.Sorfa, DavidGoncalves, Widjane Sheila FerreiraSoundy, AndrewGonzalez Garcia, JavierSouza, MargaretGorse, Christopher AndrewSpoz, AnnaGreco, GianpieroStinson, JulieGritti, EmanuelaStoian, ClaudiaGu, FengStonard, KarlieGuenther, JohnStrauss, JohannesGuerriero, MassimoStreich, JodiGuido, Giuseppe PieroStrzelecki, ArturGuinjoan, MarcSudraba, VelgaGunasekaran, KulothunganSutton, JillGurgu, ElenaSzekely, AronHa, Min-SeongTakahashi, ShoHales, AndyTakaki, ManabuHan, SuejungTampa, MirceaHardy, AnnTanaka, MasaruHarvey, NaomiTe Nijenhuis, JanHasnagas, Nikolaos D.Te Velde, VeraHeinz, MelindaTer Avest, InaHill, ShirleyTesta, GiuliaHirshler, YafitTheodorou, AnnalisaHo, Roger C.Tomaszek, KatarzynaHodgson, TimTomaz, SimoneHohl, RodrigoTomczyk, ŁukaszHonea, JoyTreslova, MarieHong, Chien-TaiUlibarri Ochoa, AinhoaHossain, SharminUnterrainer, JosefIanos, Maria GratielaUrban, WieslawIasevoli, FeliceUrbanek, ArkadiuszIngber, David A.Urchaga, José D.Ivaldi, SilviaUseche, Sergio A.Ivanova, AlyonaVagni, MoniaIzaguirre, AinhoaVaismoradi, MojtabaJahanshahi, AsgharVallely, AnneJain, PinkyVan Beest, IljaJaneczko, EmiliaVan den Noort, MauritsJean-Rémi, LapaireVan Den Tol, AnnemiekeJohnson, William FVan Overmeire, RoelJudit, OláhVarouchas, EmmanouilJulião, MiguelVelez, Anne-Lise K.Kadłubek, MartaVelez, Maria JoãoKallioniemi, ElisaVelosa, TeresaKao, Chun-ChiehVerma, ShikhaKao, Li-TingVicario, NunzioKelly, MartinaVignoli, MichelaKemper, HanVilkaite-Vaitone, NeringaKerr, JonathanVilla, ChiaraKindlon, TomVîrgă, DeliaKirchen, TwyllaVišnjić, AleksandarKlichowski, MichalWakeman, ShawneeKlimek, LiborWalkowiak, DariuszKoch, KatherineWalters, JoelKouchaki, SamanehWang, ShirleyKragness, HaleyWarms, Richard L.Krajenbrink, TrudyWayland, SarahKrasnova, Tatiana I.Weber, Kerstin M.Kubátová, JaroslavaWehmeyer, AnnKučera, ErikWeisfeld, Glenn E.Kuczyński, WojciechWilkinson, AnneKurir, Tina TičinovićWinston, Bruce E.Lai, Chiu-LinWolniak, RadosławLalueza, José LuisWood, Megan L.Langlais, MickeyXu, DingdeLautrey, JacquesZadra, CinziaLee, Hsin-Hua CathyZając-Lamparska, LudmiłaLefley, FrankZhan, TaoLeón, Samuel P.Zhang, XueyingLeón-Rubio, José MaríaZimmerman, CarlaLerma, ClaudiaZsidó, András

